# A Novel Multistage Transfer Learning for Ultrasound Breast Cancer Image Classification

**DOI:** 10.3390/diagnostics12010135

**Published:** 2022-01-06

**Authors:** Gelan Ayana, Jinhyung Park, Jin-Woo Jeong, Se-woon Choe

**Affiliations:** 1 Department of Medical IT Convergence Engineering, Kumoh National Institute of Technology, Gumi 39253, Korea; gelan@kumoh.ac.kr (G.A.); 20150555@kumoh.ac.kr (J.P.); 2 Department of Data Science, Seoul National University of Science and Technology, Seoul 01811, Korea; 3 Department of IT Convergence Engineering, Kumoh National Institute of Technology, Gumi 39253, Korea

**Keywords:** multistage transfer learning, breast cancer, classification, ultrasound, cancer cell line

## Abstract

Breast cancer diagnosis is one of the many areas that has taken advantage of artificial intelligence to achieve better performance, despite the fact that the availability of a large medical image dataset remains a challenge. Transfer learning (TL) is a phenomenon that enables deep learning algorithms to overcome the issue of shortage of training data in constructing an efficient model by transferring knowledge from a given source task to a target task. However, in most cases, ImageNet (natural images) pre-trained models that do not include medical images, are utilized for transfer learning to medical images. Considering the utilization of microscopic cancer cell line images that can be acquired in large amount, we argue that learning from both natural and medical datasets improves performance in ultrasound breast cancer image classification. The proposed multistage transfer learning (MSTL) algorithm was implemented using three pre-trained models: EfficientNetB2, InceptionV3, and ResNet50 with three optimizers: Adam, Adagrad, and stochastic gradient de-scent (SGD). Dataset sizes of 20,400 cancer cell images, 200 ultrasound images from Mendeley and 400 ultrasound images from the MT-Small-Dataset were used. ResNet50-Adagrad-based MSTL achieved a test accuracy of 99 ± 0.612% on the Mendeley dataset and 98.7 ± 1.1% on the MT-Small-Dataset, averaging over 5-fold cross validation. A *p*-value of 0.01191 was achieved when comparing MSTL against ImageNet based TL for the Mendeley dataset. The result is a significant improvement in the performance of artificial intelligence methods for ultrasound breast cancer classification compared to state-of-the-art methods and could remarkably improve the early diagnosis of breast cancer in young women.

## 1. Introduction

Breast cancer is the most common cancer in women, with approximately 2 million new cases and 685,000 deaths worldwide every year [1]. Early diagnosis decreases death from breast cancer by 40% [2,3]. Ultrasound (US) imaging is the effective modality for screening early breast cancer in women under the age of 40 years and dense breasts compared to other methods, such as mammography and biopsy, which are the current state-of-the-art breast cancer diagnosis methods [4]. However, US is not a standalone modality, in that it requires the involvement of well-trained experts in oncology and radiology, and, in most cases, a biopsy is used together with the findings from US breast imaging to determine the diagnosis results [5,6]. To improve the use of US in breast cancer diagnosis, in recent years, researchers have employed deep learning algorithms [7,8,9]. However, deep learning algorithms require a large number of breast US image training datasets to achieve high performance, which are not readily available [10]. Therefore, transfer learning (TL), which enables a model pre-trained on natural images (ImageNet) to be harnessed for segmentation, detection, or classification of US breast cancer images, has been applied to develop a relatively better performing deep learning model for breast cancer early diagnosis [11,12,13,14]. In [15], a transfer learning based breast ultrasound image classification deep learning method was proposed. In the study, the authors were able to observe the proposed method outperforming experienced radiologists. In [16], the authors proposed a transfer learning based end-to-end deep learning approach for breast ultrasound lesion recognition. The authors observed better performance using transfer learning than when using various fully convolutional networks. In [17,18], the authors implemented classification based on manually collected features, using which the authors taught the machine a feature to decide the corresponding classes. Furthermore, in [17,18], region of interest (ROI) segmentation was used prior to classification and the ROI (patches) were utilized as input for training the classifiers. In [19], the authors utilized a transfer learning method whereby an ImageNet pre-trained AlexNet network was used to classify breast ultrasound images. The authors were able to record improved performance using their proposed method. However, these conventional transfer learning (CTL) methods still could not provide the desired performance for clinical applications due to their low test accuracy and the undesired false negatives which arose when the machines were subjected to previously unseen instances [15,20,21].

Recently, multistage transfer learning (MSTL), where a model pre-trained on a large dataset (i.e., natural images) is further pre-trained on a given domain (i.e., medical images) with a relatively small dataset size compared to ImageNet, before fine-tuning it on a given target task (i.e., other medical images) with a much smaller dataset size, has become popular [22,23,24]. In MSTL, the model acquires the necessary knowledge from both the large-scale natural images and the related domains that provide the capability to effectively learn the target task with a relatively small training dataset [24]. The knowledge transferred from the first TL stage (i.e., natural image pre-trained model) helps the model to prevent overfitting, and the knowledge from the second stage (i.e., medical images) helps to learn related features in order to achieve better performance than CTL, a model pre-trained using only natural images (i.e., ImageNet) [25]. In an early work related to the application of multistage transfer learning to medical images, Samala et al. [24] utilized multistage transfer learning such that an ImageNet pre-trained model transfer-learned to classify breast digitized screen-film mammogram images, and then transfer-learned to classify digital breast tomosynthesis images. Following this, He et al. [25] published a multi-stage deep transfer learning model for the early prediction of neurodevelopment in very preterm infants via magnetic resonance imaging (MRI) images by training their model first using the brain connectome data of older children and adult autism patients. This was then followed by transfer learning on neonatal subjects’ brain connectome data pre-trained model and further transfer learning on the preterm subjects’ brain connectome dataset. Adopting this method led to an improvement in accuracy of more than 7%. In [26], first they took advantage of knowledge learned by a segmentation network from another medical imaging domain trained with a larger number of images, and then they adapted it to be able to segment general lung chest images of high quality, including COVID-19 patients. Then, using a limited dataset, composed of images from portable X-ray devices, they adapted the trained model from general lung chest X-ray segmentations to work specifically with images from these portable X-ray devices. In [27], they used multistage transfer learning to detect COVID-19 cases based on computed tomography (CT) scan images and achieved an accuracy of 86.70%, proving the capability of the model in assisting radiologists with COVID-19 diagnosis. In this study, they ensembled six ImageNet-based convolutional neural network (CNN) pre-trained models to classify CT images. The algorithm did not make use of the intermediate medical image dataset between the ImageNet dataset and CT images, only ensembling.

In this paper, to the best of our knowledge, the first MSTL algorithm applying cancer cell microscopic images for US breast cancer image classification is proposed. The proposed MSTL method involves transfer learning (TL) from an ImageNet pre-trained model to cancer cell images, which is in turn transfer learned to US breast cancer images and classifies them as either malignant or benign. The use of cancer cell images as an intermediate stage was proposed based on Samala et al. [24] because microscopic images share similar features with US images and can be acquired and used for training in a large quantity compared to other medical related images. Utilizing microscopic image data that are of sufficient quantity at the intermediate stage and employing MSTL, we show that it is possible to achieve a performance better than CTL and state-of-the-art methods for US breast cancer diagnosis. Our method paves the way for better deep learning models, pre-trained on domains similar to medical images, to be constructed as a readymade model that can be used for various medical purposes.

## 2. Materials and Methods

**Figure 1:**
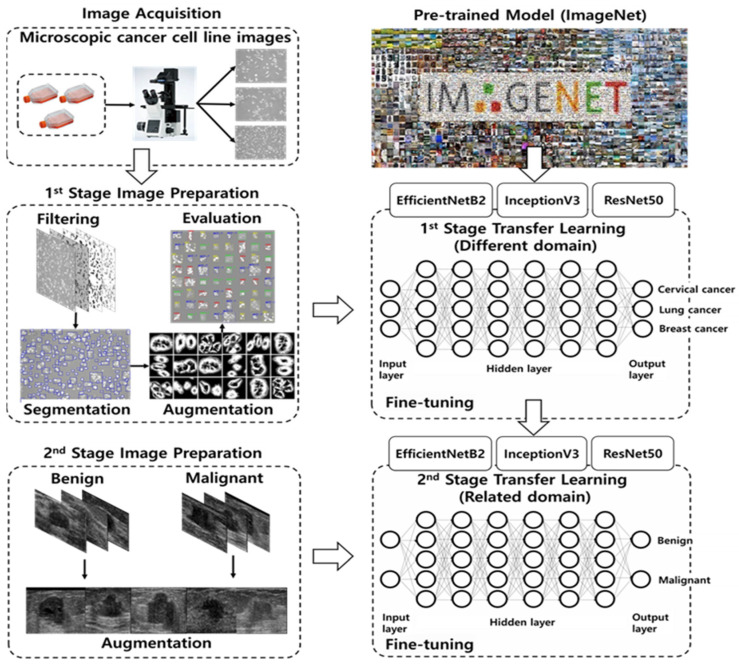
Multistage transfer learning for early diagnosis of breast cancer using ultrasound.

The proposed MSTL method involves TL from an ImageNet (dataset containing 1000 categories and 1.2 million images) pre-trained model to cancer cell line microscopic images (dataset containing three categories and 20,400 images), which is in turn used as a pre-trained model for TL on US breast cancer images (200 Mendeley and 400 MT-Small-Dataset images) to classify them as malignant or benign (Figure 1). In the first stage, we applied TL from ImageNet to cancer cell line microscopic images. This stage changes the natural image domain to a microscopic image domain by extracting more features similar to US images from the microscopic images. In the second stage, we utilized the first-stage TL as a starting point and assigned weights to the model that classifies US breast cancer images as malignant or benign. The objective of our MSTL task is to benefit from knowledge acquired through learning at different stages of TL from different image domains, using both natural (i.e., ImageNet) and microscopic images (i.e., cancer cell lines).

Given a source domain, Ds, and learning task, Ts, a target domain, Dt_,_ and learning task, Tt, transfer learning aims to help improve the learning of the target function, $f_{t}\left(.\right)$, in Dt using the knowledge in Ds and Ts [28]. This definition is used for a single-step transfer learning algorithm. However, in our case, we performed two-step transfer learning. The first-stage transfer learning involves a model trained on ImageNet (natural images) transfer-learned to classify cancer cell images. At this stage, we are interested only in changing the natural domain to the microscopic image domain. Assume that we have m training samples in the ImageNet dataset $\left\{\langle x^{1},\;y^{1}\rangle ,\ldots ,\;\langle x^{i},\;y^{i}\rangle ,\ldots ,\;\langle x^{m},\;y^{m}\rangle \right\}$ where $x^{i}$ is the *i*th input and $y^{i}$ is the corresponding label. The first-stage transfer learning takes the weights $W_{o}$ from the ImageNet pre-trained model and produces $W_{1}$ by minimizing the cross-entropy objective function in (1) [29].

(1) $$J\left(\langle W_{1},\;b|W_{0}\rangle \right)=\frac{-1}{mn}\sum_{i=1}^{m}\sum_{j=1}^{m}y^{ij}\log\left(P\langle y^{ij}|x^{ij},\;W_{0},\;W_{1},\;b\rangle \right),$$

where $\langle y^{ij}|x^{ij},\;W_{0},\;W_{1},\;b\rangle$ is the output probability of the Softmax unit [30] in the first-stage transfer learning, and b is a bias [31]. Next, assume that we have m training samples in the cancer cell line image dataset, $\left\{\langle x^{1},\;y^{1}\rangle ,\ldots ,\;\langle x^{i},\;y^{i}\rangle ,\ldots ,\;\langle x^{m},\;y^{m}\rangle \right\}$ where $x^{i}$ is the *i*th input and $y^{i}$ is the corresponding label. The second-stage transfer learning takes the pre-trained weights $W_{1}$ and produces $W_{2}$ by minimizing the following cross-entropy objective function (2):

(2) $$J\left(\langle W_{2},\;b|W_{1}\rangle \right)=\frac{-1}{m}\sum_{i=1}^{m}y^{i}\log\left(P\langle y^{i}|x^{i},\;W_{1},\;W_{2},\;b\rangle \right)+\left(1-y^{i}\right)\log\left(1-P\langle y^{i}|x^{i},\;W_{1},\;W_{2},\;b\rangle \right),$$

where $P\langle y^{i}|x^{i},\;W_{1},\;W_{2},\;b\rangle$ is the output probability of the sigmoid unit [32] in the second-stage transfer learning, and b is a bias.

### 2.1. Datasets and Pre-Processing

**Figure 2:**
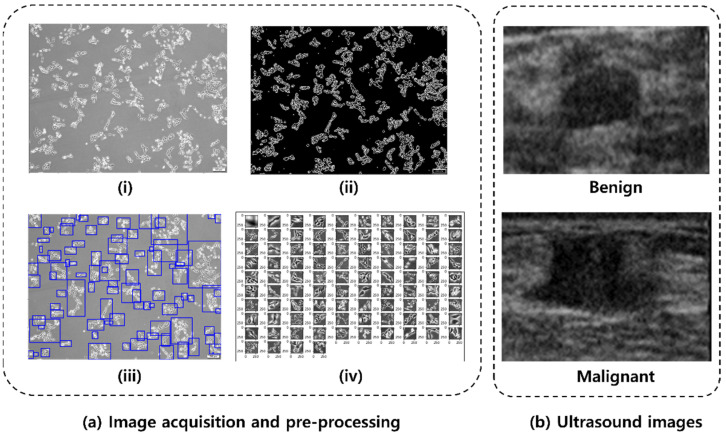
(**a**) Cancer cell images acquisition and pre-processing: (**i**) acquired HeLa cell image, (**ii**) binary image, (**iii**) image segmentation, and (**iv**) extracted image for training. (**b**) Representative Mendeley breast ultrasound images.

The cancer cell lines [33,34] for this experiment were cultured for seven days, and bright-field images were acquired every day using an inverted fluorescent microscope (IX73 with DP80, Olympus Corp., Tokyo, Japan). There were a total of three cell lines used in the experiment: HeLa (human, cervical cancer cells), MCF-7 (human, breast cancer cells), and NCI-H1299 (human, lung cancer cells), which were utilized within 6 months after receipt. All cells were purchased from the Korean Cell Line Bank (Seoul, Republic of Korea) and cultured as follows: the cell lines were cultured in high-glucose Dulbecco’s Modified Eagle Medium containing 10% fetal bovine serum and 1% penicillin streptomycin. The prepared cells were incubated at 37 °C in a humidified incubator with 5% CO_2_. Each cell line was photographed every day for 7 days after starting the cell culture, and a total number of 608 images were taken (247 images of HeLa, 149 images of MCF-7, and 212 images of NCI-H1299). To use the acquired cell images for deep learning, it is necessary to acquire a number of morphological types of cell images as they grow from the early stage to the fully grown stage. Furthermore, deep learning requires a segmentation step that distinguishes only cells present in the ROI, as cells tend to grow into groups ranging from a few to hundreds. Therefore, to improve learning efficiency and accuracy, images were pre-processed and segmented using OpenCV (version 4.5.1.48, Russia, OH, USA) and scikit-image [35] available in Python. OpenCV and scikit-image are open sources that are mainly used for real-time computer images. The colored cell images, acquired through the microscope, were converted to a grayscale image, and then converted to a binary image using adaptive thresholding [36] by OpenCV. After removing noise using the dilation function with a 2 × 2 kernel and scikit-image, the segmented image that contain only the cell body part was obtained. The processed binary image allows the identification of each cell’s contour and the creation of bounding boxes surrounding the cell. The size of the generated bounding box is proportional to the size and number of cells, and in the selected area, uninformative cells, or floating debris (the sum of width and height less than 100 pixels) were excluded from the training process. Segmented images obtained by this process were stored as independent images and used as deep learning data. The cancer cell line images acquisition process is summarized as in Figure 2a for HeLa cell line. The acquired microscopic HeLa cell line image (Figure 2a(i)) is first binarized (Figure 2a(ii)) and then subjected to segmentation (Figure 2a(iii)), which resulted in patches of HeLa cell line images (Figure 2a(iv)) for training. By segmenting 608 cell bright-field images obtained through the microscope, 6800 images of each cell line were randomly chosen to form a total of 20,400 datasets. The cancer cell line data were categorized using a 7:2:1 ratio for training, validation, and test sets (i.e., 14, 280 training, 3060 validation, and 3060 test). The training data was further augmented (i.e., rotation, width and height shift, and vertical flip) to increase the training data size to 28,560 images.

The US image data used for this study were obtained from the publicly available Mendeley dataset (https://data.mendeley.com/datasets/wmy84gzngw/1 (accessed on 8 June 2021)) composed of 250 breast US images, of which 150 are malignant cases and 100 are benign cases [37]. The dataset has been widely used in various studies [17,18,19] and is convenient to use for our purpose. We used 200 images (100 malignant and 100 benign) for preventing bias from using different sizes of data for the two classes. The 100 malignant images were picked randomly from the 150 malignant cases available in the dataset; see Figure 2 for representative benign and malignant ultrasound images. Benign tumors do not spread to other organs whereas malignant tumors spread to other organs. Augmentation (vertical flip and rotation) was applied to increase the number of training images up to 360 images [38,39]. The original images from the dataset are of different sizes, so input images were resized to be 75 × 75 pixels to avoid additional zero-padding operations. The US images were categorized using a 6:2:2 ratio for training, validation, and test sets, consecutively before the nested five-fold cross validation.

### 2.2. Convolutional Neural Network (CNN) Model

**Figure 3:**
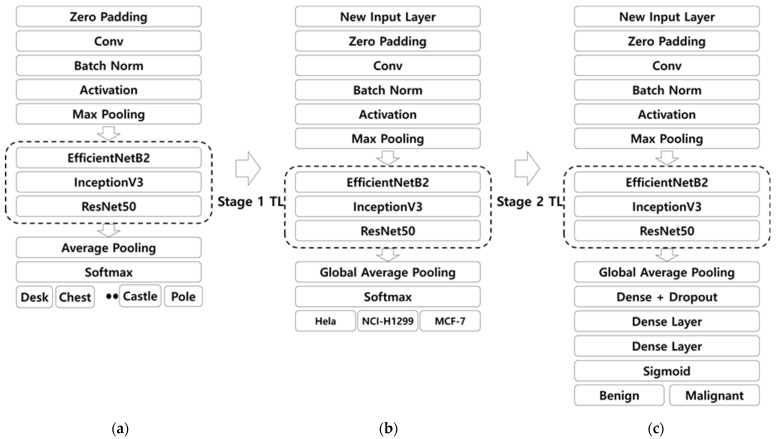
CNN models at each stage of transfer learning. (**a**) Original ImageNet pre-trained model. (**b**) ImageNet pre-trained model that is transfer-learned to cell line images. (**c**) ImageNet followed by cell line images pre-trained model that is transfer-learned to ultrasound images. Conv: Convolution; TL: Transfer Learning; Norm: Normalization.

The same protocol was utilized for all three CNN models, EfficientNetb2 [40], InceptionV3 [41], and ResNet50 [42], at each stage of transfer learning. Here, we chose the three models based on a preliminary study carried out using six pre-trained models, popularly used for classification of ultrasound breast cancer images, including AlexNet, VGG19, U-Net, InceptionV3, EfficientNetb2, and ResNet50 [11]. All the models were pre-trained on ImageNet and used as a pre-trained CNN for transfer learning on cancer cell lines. The implementation of the pre-trained model training is presented in Figure 3a, where the weights pre-trained on ImageNet were loaded using Keras. In transfer learning from the ImageNet pre-trained model to cancer cell line images in the first stage of transfer learning, only the last layer was removed and global average pooling was added, and one dense layer with Softmax was utilized, as shown in Figure 3b. We fine-tuned all the weights except the last layer from the ImageNet pre-trained model with a learning rate that decays exponentially, starting from 0.001. We employed augmentation to increase the number of cancer cell training datasets via horizontal, vertical, and rotation augmentation, which made the training data 38,080. Here, neither drop out nor regularization was a significant factor because the model showed the best performance when compared with the cases including both regularization and dropout, which implies that there was no overfitting due to the presence of large cancer cell line images. In the transfer learning from the cell line image pre-trained model to ultrasound images in the second stage of transfer learning, the dense layer was removed and replaced with three dense layers, one along with drop out [43] and lastly, the Softmax layer was replaced with a sigmoid function to give the final CNN architecture, as shown in Figure 3c. The ultrasound images were subjected to augmentation before training, which was the vertical flip and rotation that increased the number of ultrasound training image data by three-fold to make a training data size of 360 images. All the weights of the pre-trained cell line images were fine-tuned during training except the last layer. The other parameters used were the same as those of the pre-trained cancer cell line images.

### 2.3. Implementation of the Multistage Transfer Learning Method

The algorithms were executed on an RTX 3090 GPUs. The model was trained for 50 epochs at each TL stage, which was achieved after careful studying using 20–150 epochs. During the training, the learning rate was initially set at 0.001 and decayed exponentially at a decay rate of 0.96, which was the same throughout each TL stage. The training batch size [44] was set to 16. In both stages, TL was used as a fully fine-tuned model where all the weights were updated during training except the last layer.

### 2.4. Performance Measures of the Proposed Multistage Transfer Learning Method

To evaluate the proposed MSTL method, performance analysis in terms of the area under ROC curve (AUC) [45], specificity, sensitivity, and F1 measure were employed in addition to test accuracy and loss [46]. These performance metrics were evaluated by averaging over five-fold, nested cross-validation results [47]. The five-fold cross-validation divides the total dataset into five equally sized subsets that help to combat the risk of having a model that works well on training data but fails on data that it has never seen before. Finally, statistical evaluation using the *t*-test *p*-value was calculated to see the significance of the performance improvement of MSTL over CTL [48].

The area under the ROC curve (AUC) measures the entire two-dimensional area underneath the entire ROC curve. The AUC ranges from 0 to 1. A model whose predictions were 100% wrong had an AUC of 0; one whose predictions were 100% correct had an AUC of 1.

Accuracy in classification problems is the number of correct predictions made by the model over all types of predictions made given by (3)

(3) $$Accuracy=\frac{TP+TN}{TP+FP+FN+TN},$$

where, in the numerator, are correct predictions (true positives (*TP*) and true negatives (*TN*)) and in the denominator, are all predictions made by the algorithm (right as well as wrong ones), where *FP* is false positive and *FN* is false negative. 

Specificity (4) is a measure that tells us what proportion of patients, who did not have cancer, were identified by the model as non-cancerous.

(4) $$Specificity=\frac{TN}{TN+FP},$$

Sensitivity (5) is a measure that tells us what proportion of patients, who actually had cancer, were correctly diagnosed by the algorithm.

(5) $$Sensitivity=\frac{TP}{TP+FN},$$

The *F*1 measure (6) is the harmonic mean of precision and recall, and the highest possible value of an *F*1 measure is 1, indicating perfect precision and recall, and the lowest possible value is 0 if either the precision or recall is zero.

(6) $$F1=\frac{TP}{TP+\frac{1}{2}\left(FP+FN\right)},$$

## 3. Results

### 3.1. The Multistage Transfer Learning Performance

The average performance results of the proposed MSTL algorithm over 5-fold cross validation (see Table S1 under SI 1 for each fold cross validation results) for the EfficientNetB2, InceptionV3, and ResNet50 pre-trained models are presented in Table 1. For each CNN model, three experiments were conducted using three optimizers, stochastic gradient descent (SGD), Adam, and Adagrad [38]. Among the model combinations tested, it was observed that ResNet50 with the Adagrad optimizer provided the highest test accuracy of 99 ± 0.612%, the smallest loss of 0.03, as well and the highest AUC, specificity, sensitivity, and F1 measure of 0.999, 0.98, 1, and 0.989, respectively (see Figures S1 and S2 under Supplementary Materials for MSTL learning curves of each model). Generally, ResNet50 performed best with an average test accuracy of 98% averaged over the three optimizers, followed by InceptionV3 with a test accuracy of 92%, and EfficientNetB2 with 90% test accuracy.

### 3.2. Comparison of the Proposed Multistage Transfer Learning Method with Conventional Transfer Learning Methods to Classify Ultrasound Images

**Figure 4:**
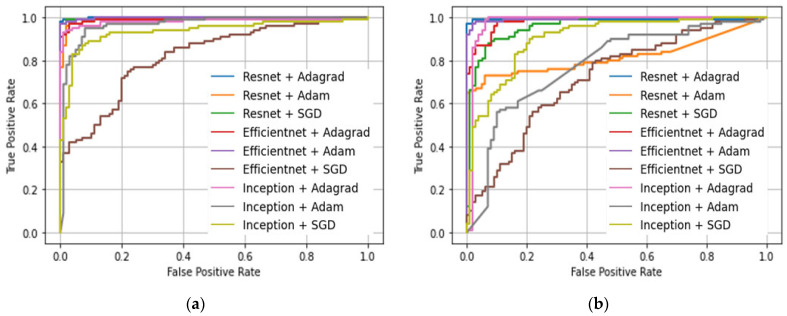
ROC curves comparison. (**a**) Multistage transfer learning. (**b**) Conventional transfer learning. SGD: Stochastic gradient descent.

A comparison of the proposed MSTL, used to classify US breast cancer images, against the CTL, which is based on ImageNet pre-trained model, was carried out using three ImageNet pre-trained models with three optimizers, as shown in Table 1. From the averaged accuracy measure over the three optimizers, MSTL provided better performance than CTL. The ROC curves comparison of the CTL with the proposed MSTL is shown in Figure 4, which shows that the proposed MSTL achieved a better ROC curve compared to the CTL. We have also calculated the *t*-test *p*-value to measure the significance of the improvement due to the use of cancer cell images in the second stage of our MSTL in order to compare it against the CTL. Here, we considered all the average 5-fold cross-validation accuracy results from all CNN and optimizer combinations. The resulting *p*-value was 0.01191 (i.e., a probability of 1.191% that the improvement in performance from using MSTL will be false), which is less than the 0.05 (i.e., 5%) standard significance cut-off *p*-value [48]. This shows that our MSTL made a significant improvement in the performance of classifying US breast cancer images when compared to the CTL. Moreover, the learning process in multistage transfer learning is more stable than the conventional transfer learning. This can be depicted by observing the loss amounts in each model for MSTL and CTL, as shown in Table 1. The loss values in MSTL are smaller and smoother than those of the CTL. For instance, the CTL trained InceptionV3-Adam has a loss of as high as 9.57, whereas the same model using MSTL has a small loss of 0.292, which shows a huge loss difference. The lowest loss using CTL is 0.084 whereas using MSTL it is 0.03, which shows that MSTL has a lower loss than CTL.

### 3.3. The Effect of Optimizers and Pre-Trained Base Models

**Figure 5:**
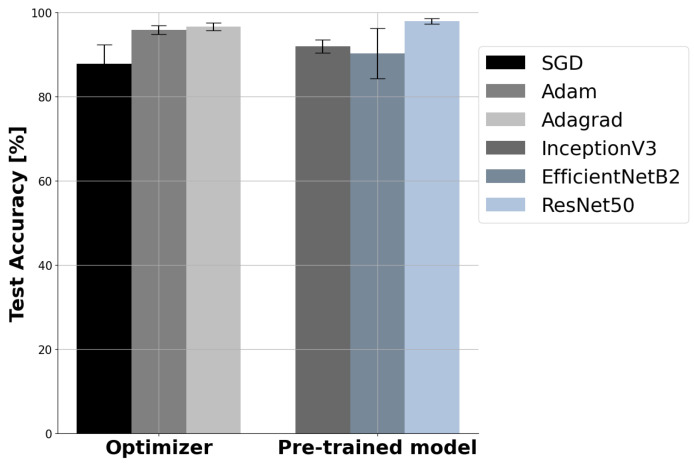
(**Left**) The effect of optimizer choice on the performance of multistage transfer learning. (**Right**) The effect of CNN model choice on the performance of multistage transfer learning. SGD: stochastic gradient descent.

Figure 5(Left) shows that optimizer choice affects performance. Among the three optimizers (SGD, Adam, and Adagrad) used, Adagrad is the best optimizer in MSTL for the classification of breast ultrasound images, with the highest average accuracy of 96.67 ± 1.8%, followed by Adam, with average accuracy of 95.83 ± 2%, whereas SGD is the least best, with average accuracy of 87.83 ± 8.9%. This might be due to Adagrad’s performance superiority for sparse datasets and datasets with missing samples, which is true in our case where a small dataset size was utilized. Even though SGD is fast and simple, it gets stuck at a local minimum, whereas Adam is well suited for big datasets [38]. Based on evaluations carried out, the use of different CNN models resulted in different performances. ResNet50 outperformed InceptionV3 and EfficientNetB2 models in terms of almost all of the performance measures used in this study, as depicted in Table 1. Figure 5(Right) describes the effect of CNN model choice on performance in terms of accuracy, where ResNet50 has the highest accuracy with the lowest standard deviation (98 ± 1%) compared to the InceptionV3 (92 ± 3.1%) and EfficientNetB2 (90.3 ± 9.7%) models.

### 3.4. Comparison with Published Works

There are a few published works on the application of TL to classify US breast cancer images. A comparison of the proposed method with previous works using the same dataset is presented in Table 2. The proposed MSTL showed the best performance compared to all published papers using the Mendeley dataset including, Acevedo et al. [17], Zeebaree et al. [18], and Guldogan et al. [19], with accuracies of 94%, 95.4%, and 97.4%, respectively. In [17,18], the authors implemented classification based on manually collected features, which is how the authors taught the machine a feature to decide corresponding class, whereas in our case, we carried out an end-to-end deep learning where the model itself learns the features of each class and decides on the corresponding class using the rich capability of CNNs. Furthermore, in [17,18], ROI segmentation was used prior to classification and the ROI (patches) were utilized as input for training the classifiers, whereas in our case, rather than carrying out ROI segmentation, we utilized the image as it is. This results in the merit of having a model that is fast and not computationally complex. In [19], the authors utilized a conventional transfer learning method whereby an ImageNet pre-trained AlexNet network is used to classify breast ultrasound images. In our case, we used a multistage transfer learning method whereby additional transfer learning using cancer cell lines was carried out on top of ImageNet prior to transfer learning to classify breast ultrasound images. Due to the fact that cancer cell line images possess resemblance to ultrasound images, superior transfer learning was achieved using our method on the same dataset when compared to [19].

### 3.5. Experiment with MT-Small-Dataset

To further study the performance of the proposed multistage transfer learning meth-od, we utilized another ultrasound image dataset, MT-Small-Dataset, a dataset derived from the breast ultrasound images (BUSI) dataset [49]. The MT-Small-Dataset (https://www.kaggle.com/mohammedtgadallah/mt-small-dataset (accessed on 10 September 2021)) is a collection of 400 breast ultrasound images with tumors and their 400 ground truth images [50], which is composed of 200 benign and 200 malignant breast images. We used the same process as in the case of Mendeley dataset. The dataset is made up of breast ultrasound images from a variety of women aged between 25 and 75 years old, acquired by the LOGIQ E9 ultrasound and LOGIQ E9 Agile ultrasound systems, at Baheya Hospital for Early Detection and Treatment of Women’s Cancer, Cairo, Egypt. Based on the study with the MT-Small-Dataset, our best multistage transfer learning method (ResNet50 with Adagrad) achieved a test accuracy of 98.7 ± 1.1%, AUC of 0.98, F1-score of 0.966, sensitivity of 0.974, and specificity of 0.968 in classifying the images as benign or malignant.

## 4. Discussion

**Figure 6:**
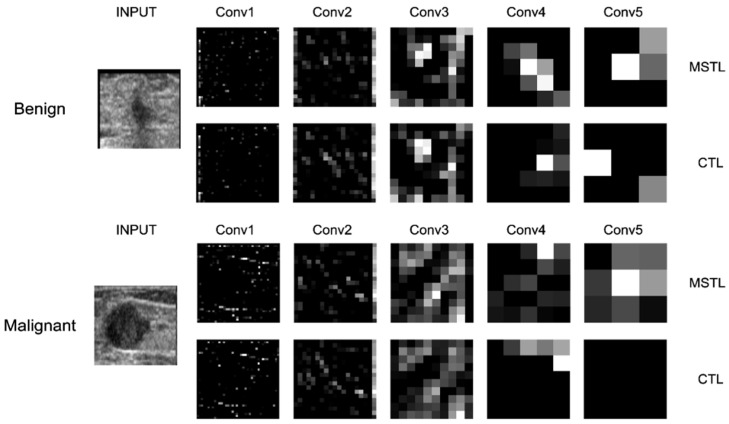
Feature extraction comparison via feature visualization of the five convolution layers of ResNet50 with the Adagrad optimizer for MSTL and CTL. Conv: convolution; MSTL: multistage transfer learning; CTL: conventional transfer learning.

The significance of this study is to show that with the use of MSTL via natural images, which are readily available, and microscopic images that can be acquired in large amounts, a high-performance CNN model can be developed. Our MSTL model has the advantage of learning image features from the large ImageNet dataset with millions of images at the first stage of TL and from the cancer cell line images that enables the CNN model to learn more details about features similar to ultrasound images at the second stage of TL. With all these features, learned from both natural image data and microscopic image data, the proposed MSTL method achieved high accuracy in classifying US breast cancer images as benign or malignant. The experiments revealed that the proposed MSTL method outperformed the CTL methods pre-trained only on ImageNet. The CTL models are pre-trained on vastly available natural images, and when transfer-learning is applied to a small number of US breast cancer datasets, the models overfit the data and do not perform very well when subjected to new instances of data. Additionally, because the domains of the source and target images are different, the features learned from the pre-trained models on natural images will be limited to generalize for medical images that are different from natural images. In Figure 6, we provide feature visualization for the five convolution layers of representative ResNet50 model with the Adagrad optimizer to show how our MSTL method improved the feature extraction activity for classifying the US images as benign/malignant when compared to CTL. The CTL performed well in recognizing edge features (the first two convolutions), as expected, but not in textural structures extraction due to the fact that it was pre-trained only on natural images [51]. In contrast to this, the MSTL performed well in extracting features from edge structures (the first two convolutions) as well as texture structures (the last two convolutions) by leveraging the knowledge acquired by being pre-trained both on natural and microscopic images (see the bright features in Figure 6). Generally, our method paves the way for better deep learning models, pre-trained on medical images, to be constructed as a readymade model that can be used for various medical purposes.

To the best of our knowledge, this is the first attempt to employ MSTL to classify US breast cancer images. There are some limitations in this study that should be acknowledged. In our experiments, we selected only three models and three optimizers and kept other parameters such as learning rate, training batch size, and augmentation constant for all cases to enable a fair comparison. However, investigations in the future should consider more pre-trained models and optimizers other than those used in this study. It is important to note that the models and optimizers were selected by considering state-of-the-art models and optimizers based on our previously published work [10]. Additionally, varying the different parameters and conducting further studies to determine the effects of other hyperparameters should be considered. Moreover, we were able to see from this study that the use of cancer cell line microscopic images at the second stage of transfer learning improved the performance of US breast cancer image classification by providing knowledge of features more similar to US images than the natural images. However, experiments were not carried out to determine the effects of using other types of cancer cell line images, as well as varying the quantities of cancer cell line images. Further studies should be conducted to investigate the effects of other types of cancer cell lines, as well as whether the quantity of cancer cell images used has an effect. Finally, this study utilized only the publicly available Mendeley and MT-Small-Dataset US breast cancer image datasets to produce the results reported in this paper. Future studies should consider using a range of datasets.

In conclusion, we developed a multistage transfer learning method using natural and cancer cell line images to distinguish between benign and malignant ultrasound breast cancer images. To do so, features learned from the large natural image dataset (i.e., ImageNet) and the cancer cell line microscopic image dataset were transfer-learned for the classification of ultrasound breast cancer images through multistage transfer learning. Our approach classified breast cancer with a test accuracy of 99 ± 0.612% on the Mendeley dataset and 98.7 ± 1.1% on the MT-Small-Dataset. This study demonstrates that large cancer cell line image dataset collected via microscope are useful for developing high performance early breast cancer diagnosis methods using ultrasound, alleviating the need for finding large ultrasound data sets for the realization of high-performance deep learning models. The proposed system has a huge impact on the diagnosis of early breast cancer, which is crucial for decreasing the mortality rate of breast cancer. Furthermore, it has the potential to save patients from unnecessary biopsies and improve clinical decision-making. 

## Figures and Tables

**Table 1:** The averaged performance results over 5-fold cross validation of the proposed multistage transfer learning and its comparison against conventional transfer learning. TL: transfer learning; CNN: convolutional neural network; AUC: area under ROC curve, Avg.: average; CTL: conventional transfer learning; MSTL: multistage transfer learning; SGD: stochastic gradient descent.

TL Type	CNN	Optimizer	AUC	F1 Measure	Specificity	Sensitivity	Loss	Test Accuracy (%)	Avg. Test Acc. (%)
CTL method	InceptionV3	SGD	0.903	0.833	0.87	0.80	0.412	83.50 ± 5.491	83
		Adam	0.778	0.605	0.66	0.75	9.570	70.50 ± 6.085	
		Adagrad	0.976	0.967	1	0.93	0.195	96.49 ± 2.091	
	EfficientNetb2	SGD	0.717	0.664	0.71	0.61	0.644	66.00 ± 1.895	83
		Adam	0.993	0.948	0.98	0.90	0.194	93.99 ± 4.726	
		Adagrad	0.980	0.904	0.98	0.81	0.300	89.50 ± 2.709	
	ResNet50	SGD	0.960	0.902	0.90	0.91	0.296	90.50 ± 2.850	89
		Adam	0.817	0.698	0.66	0.97	0.117	81.50 ± 10.216	
		Adagrad	0.989	0.974	0.97	0.98	0.084	97.50 ± 2.165	
The proposed MSTL method	InceptionV3	SGD	0.935	0.873	0.83	0.94	0.458	88.50 ± 3.758	92
		Adam	0.967	0.930	0.94	0.92	0.292	93.00 ± 2.291	
		Adagrad	0.981	0.945	0.95	0.94	0.208	94.50 ± 0.935	
	EfficientNetB2	SGD	0.820	0.762	0.77	0.76	0.606	76.50 ± 3.409	90
		Adam	0.998	0.980	0.98	0.98	0.067	97.99 ± 1.249	
		Adagrad	0.992	0.965	0.97	0.96	0.207	96.50 ± 1.274	
	ResNet50	SGD	0.995	0.985	0.99	0.98	0.065	98.50 ± 1.118	98
		Adam	0.986	0.964	0.96	0.97	0.216	96.49 ± 1.000	
		Adagrad	0.999	0.989	0.98	1	0.030	99.00 ± 0.612	

**Table 2:** Comparison of the proposed multistage transfer learning method with state-of-the-art ultrasound breast cancer classification methods. SVM: support vector machine; ANN: artificial neural network; AUC: area under ROC curve.

Paper	CNN	Application	Image Dataset	Train-/Validation/Test Size	Performance
Acevedo et al. [17]	SVM	Classification	Mendeley	250 images (150 malignant and 100 benign)	Accuracy = 94%
Zeebaree et al. [18]	ANN	Segmentation Classification	Mendeley	50 images for training (25 from each class)	Accuracy = 95.4%
Guldogan et al. [19]	AlexNet	Classification	Mendeley	250 images (150 malignant and 100 benign): 85% training; 15% test	Specificity = 1 Sensitivity = 0.957 Accuracy = 97.4%
The proposed MSTL method	ResNet50	Classification	Mendeley	200 images (100 from each class): 60% training; 20% validation; 20% test	AUC = 0.999 F1 measure = 0.989 Specificity = 0.98 Sensitivity = 1 Accuracy = 99%

## Data Availability

In this study, we used publicly available breast ultrasound images, the Mendeley ultrasound dataset (https://data.mendeley.com/datasets/wmy84gzngw/1 (accessed on 8 June 2021)) and the MT-Small-Dataset (https://www.kaggle.com/mohammedtgadallah/mt-small-dataset (accessed on 10 September 2021)). The cancer cell line images can be made available for reasonable requests by contacting the corresponding authors.

## Supplementary Materials

The following are available online at https://www.mdpi.com/article/10.3390/diagnostics12010135/s1. Table S1: Five-fold cross validation comparison results of each model for the conventional as well as multistage transfer learning methods, Figure S1: Individual learning curves for the multistage transfer learning models over 5-fold cross-validation, Figure S2: Multistage transfer learning models training and validation accuracy and loss curves.
