# Supplementary material for: A Novel Multistage Transfer Learning for Ultrasound Breast Cancer Image Classification
Source: Diagnostics (Basel). 2022 Jan 6;12(1):135. doi: 10.3390/diagnostics12010135 (PMC8775102; doi:10.3390/diagnostics12010135)
Supplement: Supplementary file 1 [file diagnostics-12-00135-s001.zip › diagnostics-1501165-supplementary.pdf]

diagnostics

MDPI

Supplementary Materials: A novel multistage transfer learning for ultrasound breast cancer image classification

Gelan Ayana, Jinhyung Park, Jin-Woo Jeong * and Se-woon Choe *

Table S1. Five-fold cross validation comparison results of each model for the conventional as well as multistage transfer learning methods.

Transfer learning type	CNN	Optimizer	Five-fold cross validation test accuracy (%)					
			Fold 1	Fold 2	Fold 3	Fold 4	Fold 5	Average
ImageNet based CTL	Incep-tionV3	SGD	97.50000238	72.50000238	80.00000119	92.50000119	75	83.50000143
		Adam	75	72.50000238	82.49999881	50	72.50000238	70.50000072
		Adagrad	100	97.50000238	89.99999762	94.99999881	100	96.49999976
	Efficient-NetB2	SGD	69.99999881	67.50000119	60.00000238	64.99999762	67.50000119	66.00000024
		Adam	100	94.99999881	77.49999762	97.50000238	100	93.99999976
		Adagrad	92.50000119	82.49999881	94.99999881	92.50000119	85.00000238	89.50000048
	ResNet50	SGD	92.50000119	92.50000119	87.5	97.50000238	82.49999881	90.50000072
		Adam	52.49999762	67.50000119	97.50000238	97.50000238	92.50000119	81.50000095
		Adagrad	100	89.99999762	100	97.50000238	100	97.5
The proposed MSTL	Incep-tionV3	SGD	97.50000238	97.50000238	85.00000238	80.00000119	82.49999881	88.50000143
		Adam	92.50000119	85.00000238	97.50000238	97.50000238	92.50000119	93.00000191
		Adagrad	94.99999881	92.50000119	94.99999881	97.50000238	92.50000119	94.50000048
	Efficient-NetB2	SGD	67.50000119	75	72.50000238	80.00000119	87.5	76.50000095
		Adam	100	94.99999881	100	94.99999881	100	97.99999952
		Adagrad	100	97.50000238	92.50000119	94.99999881	97.50000238	96.50000095
	ResNet50	SGD	100	100	94.99999881	97.50000238	100	98.50000024
		Adam	94.99999881	100	94.99999881	97.50000238	94.99999881	96.49999976
		Adagrad	100	97.50000238	100	97.50000238	100	99.00000095

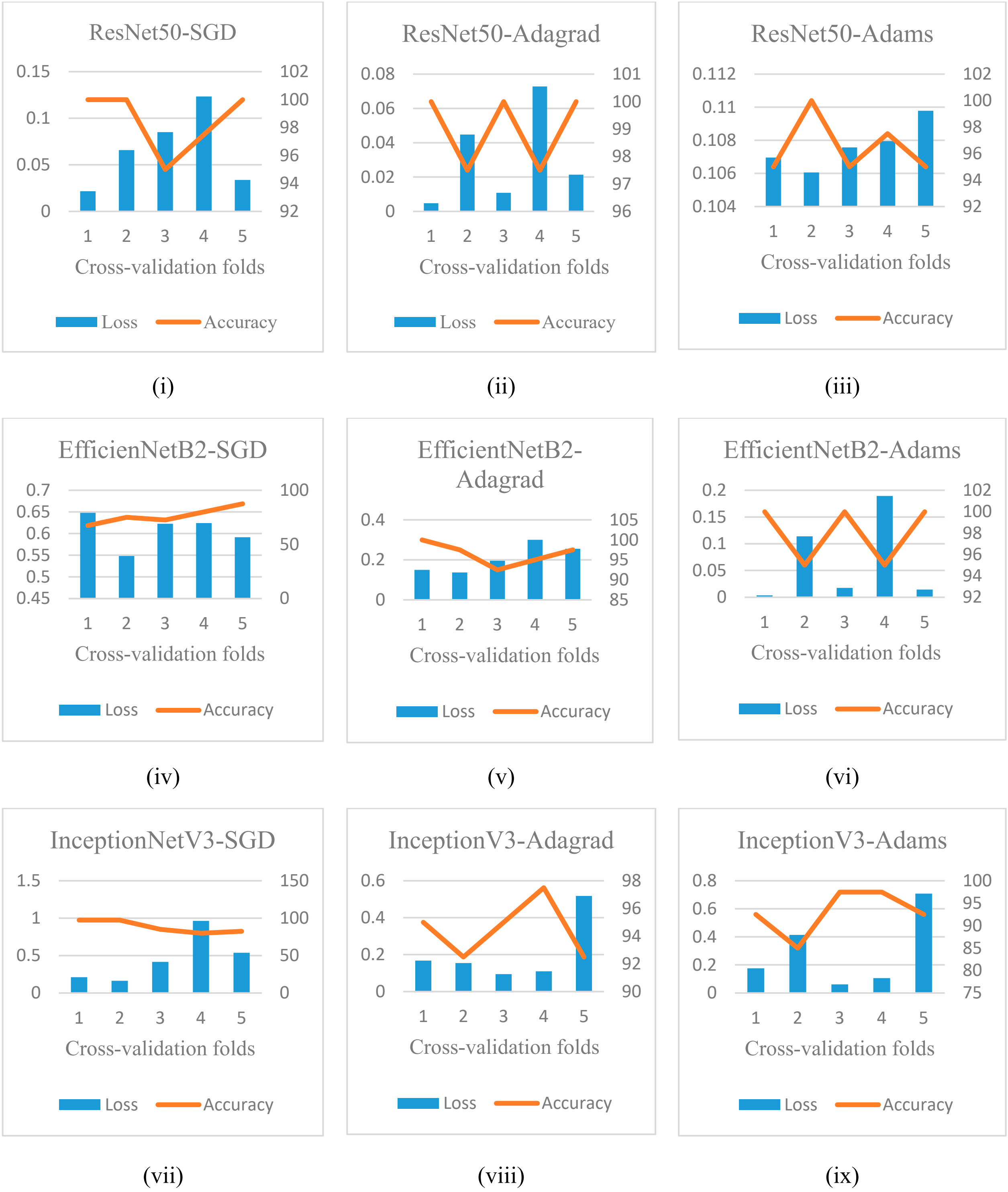

Figure S1. Individual learning curves for the multistage transfer learning models over 5-fold cross-validation. (i) ResNet50-SGD (ii) ResNet50-Adagra (iii) ResNet50-Adam (iv) EfficientNetB2-SGD (v) EfficientNetB2-Adagrad (vi) EfficientNetB2-Adam (vii) InceptionNetV3-SGD (viii) InceptionNetV3-Adagrad (ix) InceptionNetV3-Adam.

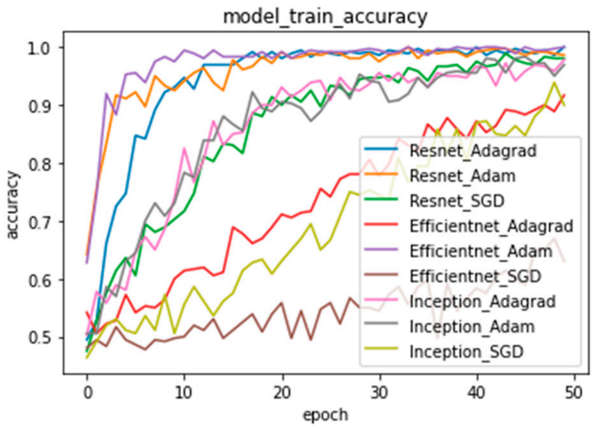

(i)

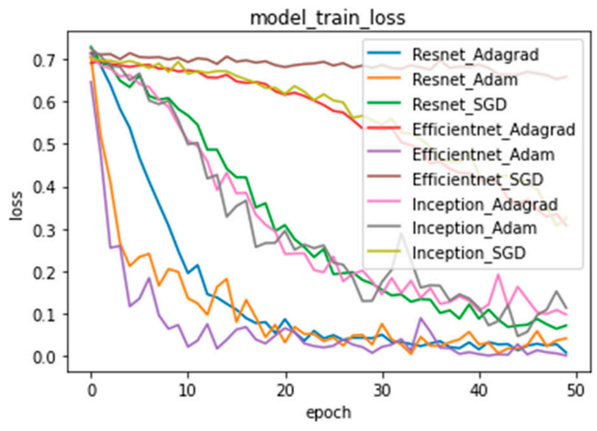

(ii)

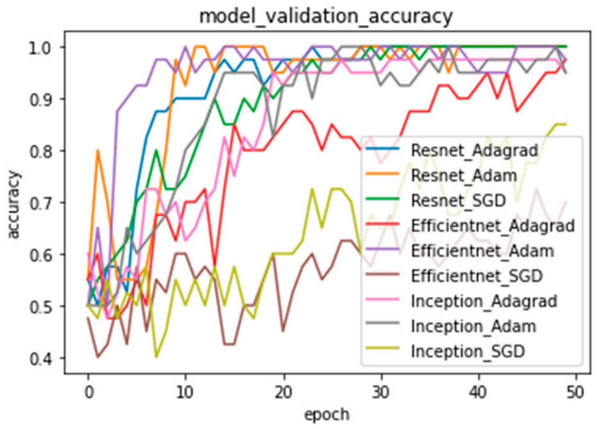

(iii)

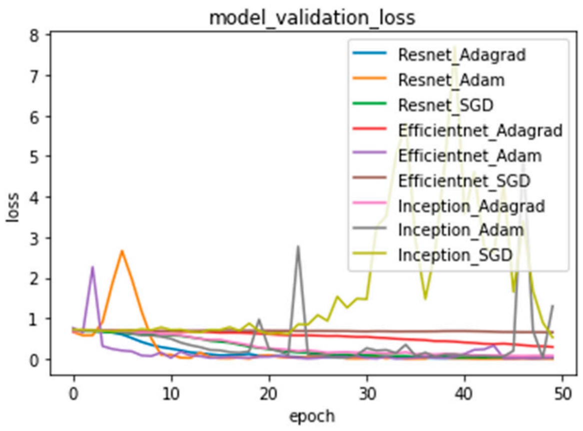

(iv)

Figure S2. Multistage transfer learning models training and validation accuracy and loss curves. (i) Training accuracy curve (ii) Training loss curve (iii) Validation accuracy curve (iv) Validation loss curve.
